# Invasive pulmonary and central nervous system aspergillosis in a child: A case report and literature review

**DOI:** 10.1097/MD.0000000000037160

**Published:** 2024-02-09

**Authors:** Dongmei Meng, Yingxue Zou, Jiao Li, Jia Zhai, Run Guo, Xingnan Jin

**Affiliations:** a Department of Pneumology, Tianjin Children’s Hospital/Tianjin University Children’s Hospital, Tianjin, China; b Department of Infectious Disease, Tianjin Children’s Hospital/Tianjin University Children’s Hospital, Tianjin, China.

**Keywords:** central nervous system, intrathecal amphotericin B, Invasive aspergillosis, metagenomic sequencing

## Abstract

**Rationale:**

Children with haematological malignancies have a higher risk of developing aggressive pulmonary aspergillosis and a higher mortality rate. The most common site of extrapulmonary aspergillosis in children is the central nervous system (CNS), and the death rate is higher when CNS is affected. Therefore, early diagnosis and treatment of invasive aspergillosis are essential for reducing mortality.

**Patient concerns:**

We report a case of an 8-year-old girl with acute lymphoblastic leukaemia who developed invasive pulmonary aspergillosis complicated by CNS aspergillosis. *Aspergillus* was confirmed by metagenomic sequencing of pathogenic microorganisms.

**Diagnoses:**

Invasive pulmonary and central nervous system aspergillosis.

**Interventions:**

The patient was treated with combined systemic antifungal agents (voriconazole and liposomal amphotericin B) and intrathecal injection of amphotericin B.

**Outcomes:**

The treatment was well tolerated and resulted in remarkable clinical and radiological head improvements.

**Lessons:**

Invasive aspergillosis has a high mortality rate and requires early diagnosis and treatment. Pathogenic microbial metagenomic sequencing is a convenient method to assist in the early diagnosis of aspergillosis. Voriconazole is the drug of choice for the treatment of invasive aspergillosis. When CNS aspergillosis occurs, it can be combined with other systemic antifungal drugs and intrathecal injection of amphotericin B.

## 1. Introduction

*Aspergillus* remains an important cause of life-threatening infections in immunocompromised patients. There are 3 main types of aspergilloses: invasive aspergillosis (IA), chronic aspergillosis, and allergic aspergillosis.^[1]^ IA can be fatal in immunodeficient individuals. Children with haematological malignancies have a higher risk of developing invasive pulmonary aspergillosis (IPA) and a higher mortality rate.^[2–4]^ The central nervous system (CNS) is the most common site of extrapulmonary aspergillosis in children.^[5]^ According to previous reports, the probability of CNS involvement is 9% to 39%, and the mortality rate is >88%.^[4,5]^ Therefore, early diagnosis and treatment are essential for reducing mortality.

Here, we report a case of an 8-year-old girl with acute lymphoblastic leukaemia (ALL) who had IPA complicated by CNS aspergillosis. The patient was treated with a combination of systemic antifungal therapy and intrathecal injection of amphotericin B. She presented with significant clinical and radiological improvement, and no complications related to drug treatment were reported.

## 2. Case presentation

An 8-year-old girl with a known history of ALL was admitted to our hospital on November 11, 2020 for fever and cough that had persisted for more than 1 month. ALL (B-cell type) was diagnosed in another hospital 5 years previously, and the bone marrow achieved complete remission after VDLD (vincristine, daunorubicin, pegaspargase, dexamethasone) induction chemotherapy and CAM (cyclophosphamide, cytarabine, and 6-mercaptopurine) consolidation chemotherapy according to the China Children’s Tumour Cooperation Group (CCCG)-ALL-2015 regimen. Two months prior, she was admitted to another hospital again because of a pale face and abdominal discomfort. After bone marrow puncture, she was diagnosed with ALL (relapsed, B-cell type, CRLF2 mutation positive). Induction chemotherapy with VILP (vincesine, idarubicin, pegaspargase, and prednisone) was administered according to the relapsed ALL-CCCG-2017 regimen. During the period of VILP induction chemotherapy in the other hospital more than 1 month prior, the child developed neutrophil deficiency (neutrophils was 0.01 × 10^9^/L), fever (body temperature of up to 40°C), and paroxysmal vocal cough. Lung computed tomography (CT) showed infectious lesions in both lungs, and the result of the blood galactomannan (GM) test was 2.04 (positive: single I > 0.7 or 2 times I > 0.5, based on enzyme-linked immunoassay). The patient received anti-infective treatment of “Meropenem 20 mg/kg q8h × 17 days, voriconazole 0.2 g q12h × 9 days, and Compound sulfamethoxazole 0.96 g qd × 4 days”; however, her condition did not improve, and she was admitted to our hospital.

Her family history was unremarkable, and she had no history of an infectious disease. She received all vaccinations as scheduled. On admission, her temperature was 37.1°C, pulse rate was 100/min, respiration rate was 20/min, and blood pressure was 100/60 mm Hg (1 mm Hg = 0.133 kPa). The pharynx was congested, and the bilateral tonsils were mildly swollen with no exudation. Symmetrical thorax was observed on both sides, with normal fluctuations, and fine rales were heard in both the lungs. Neurological examination results were unremarkable. No other remarkable abnormalities were observed.

**Figure 1.**
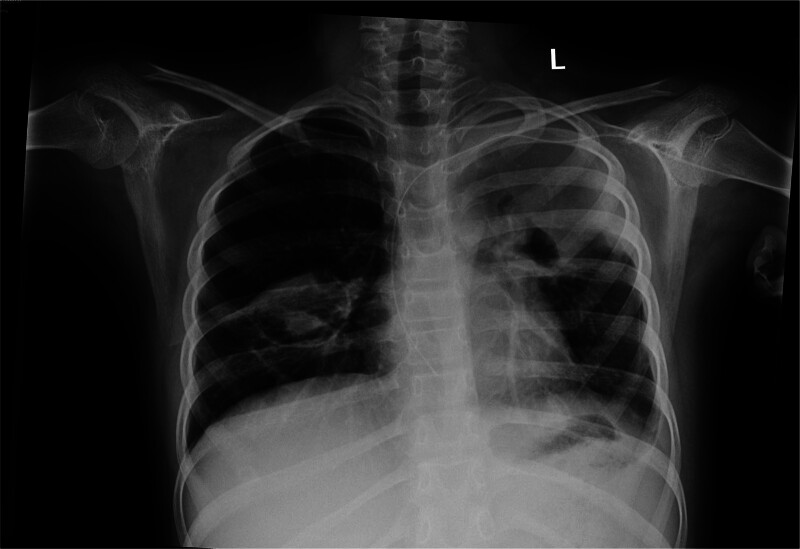
Chest radiography showed that both lungs had high-density flaky shadows, and the right lung had cavities at the lesion site.

**Figure 2.**
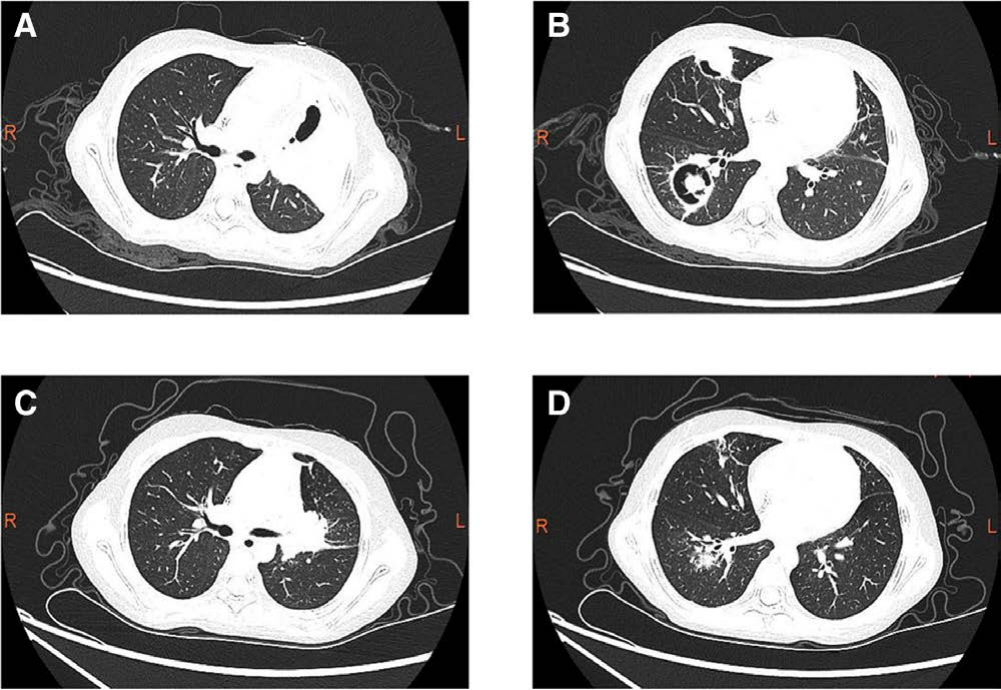
Chest CT. (A) Flaky high-density shadows with cavities in the left upper lobe. (B) Two cavitating lung nodules with “crescent sign” in the inferior lobe of right lung. (C, D) 1 month after discharge showed that the lesions were more significantly reduced. CT = computed tomography.

Initial laboratory studies showed a white blood cell count of 11.24 × 10^9^/L; absolute neutrophil count of 8.9 × 10^9^/L; hemoglobin level of 101 g/L; platelet count of 237 × 10^9^/L; C-reactive protein concentration of 145.2 mg/L (normal reference, 0–8 mg/L); procalcitonin concentration of 1.21 ng/mL (normal reference, 0–0.05 ng/mL); erythrocyte sedimentation rate of 91 mm/h (normal reference, 0–20 mm/h); and 1, 3-β-D glucan level of 163.9 pg/mL (normal reference, 0–60 pg/mL). The patient had normal renal function test results and normal liver enzyme levels. Immunoglobulin levels and flow cytometry were normal. Blood cultures and tuberculous T cells results were negative. Electrocardiogram and echocardiogram showed no abnormalities. Chest radiography showed high-density flaky shadows in the both lungs and cavities at the lesion site in the right lung (Fig. 1). Chest CT showed that both the lungs were scattered with nodular, flaky, and cord-like high-density shadows, and some cavities were visible (Fig. 2). *Aspergillus flavus (A. flavus*) was detected by metagenomic sequencing of pathogenic microorganisms in 2 batches of alveolar lavage fluid (1094 reads in the first test and 158 reads in the second test).

**Figure 3.**
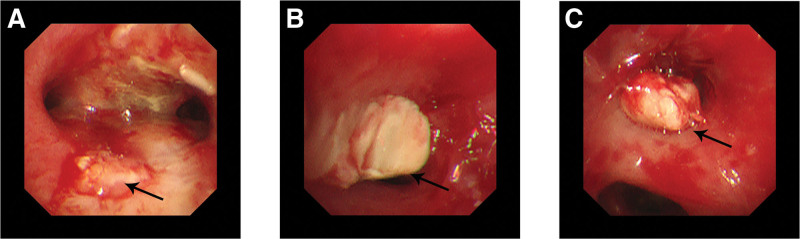
Fibreoptic bronchoscopy on day 7 (A, B) and 14 (C) after admission. (A, B) Showed growth of granulation tissue in the lower left lobe. (C) Showed a white neoplasm in the left upper lobe bronchus opening to block the airway.

After admission, the patient was administered cefoperazone sodium and sulbactam sodium 180 mg/kg q8h, linezolid 10 mg/kg q8h, voriconazole 8 mg/kg q12h, and methylprednisolone 2 mg/kg. On the second day of admission, her temperature was normal. Fibreoptic bronchoscopy on the seventh day of admission showed inflammation of the bronchial lining (lower trachea and carina) and growth of granulation tissue in the lower left lobe (Fig. 3). On the 14th day of admission, the second fibreoptic bronchoscopy showed a white neoplasm in the left upper lobe bronchus opening that blocked the airway (Fig. 3). On the 15th day of admission, the cough was relieved; however, on the 26th day after admission, the patient developed low fever again, with the highest body temperature of 37.9°C. On the 29th day of admission, a protruding mass was observed on the left side of the top of the skull, approximately 6 × 3 cm in size, which was cystic and painful when touched. Cranial B-ultrasonography showed that the affected area of the scalp was 31 × 4 mm in size, with spot-like strong echoes seen inside, suggesting a subcutaneous mass hematoma on the head. Blood coagulation and platelet count were normal. After 72 hours of cold compression, a hot compression was applied to the mass. On the 31st day of admission, her body temperature was normal, and the mass at the top of the skull had reduced slightly. reexamination of blood GM test was 0.09 and 1, 3-β-D glucan was 27.5 pg/mL before discharge. The patient was discharged on December 12, 2020. After discharge, the patient was prescribed voriconazole 0.2 g q12h (8 mg/kg q12h) orally.

One day after discharge, the patient had fever again, with the highest body temperature of 38.5°C. She had a fever daily and continued to cough. Six days after discharge, her family found progressive enlargement of the mass on the left side of the cranial apex, and she was readmitted to the hospital on December 23, 2020. After readmission, a physical examination showed a protruding mass on the left side of the cranial apex, approximately 6.5 × 5.5 × 1.5 cm in size, which was cystic and accompanied by pain when the contact pressure was obvious. Wet rales were audible in both the lungs. Neurological examination results were unremarkable.

**Figure 4.**
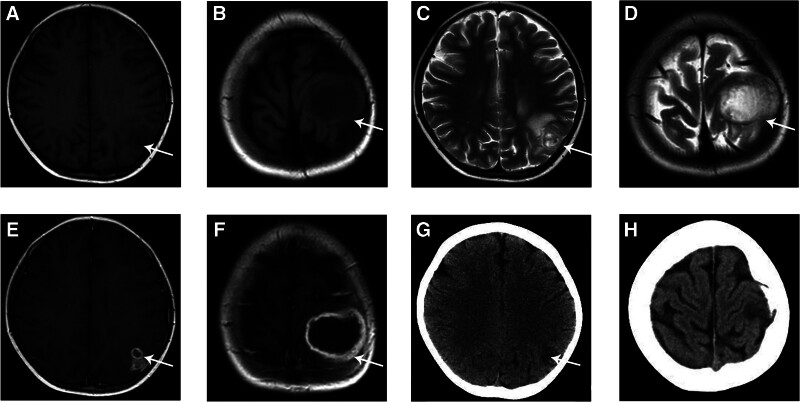
Head MRI (A, B) showed multiple longer T1 in the left parietal lobe. (C, D) showed multiple longer T2 in the left parietal lobe. (E, F) The enhanced scan showed multiple abnormal signal lesions in the left parietal lobe with circular enhancement-the possibility of abscess formation. Head CT (G, H) 1 month after discharge showed that the lesions were more significantly reduced. CT = computed tomography.

Head magnetic resonance imaging (MRI) showed multiple uneven and slightly longer T1 and long T2 signal shadows in the left parietal lobe. The enhanced scan showed multiple abnormal signal lesions in the left parietal lobe with circular enhancement, and the edge of the subcutaneous mass on the left top with obvious enhancement. We considered the possibility of abscess formation (Fig. 4). reexamination of blood GM test was 0.1 and 1, 3-β-D glucan was 37.5 pg/mL. Regarding bone marrow cell morphology, there were 3 lines of bone marrow hyperplasia after ALL treatment. The cerebrospinal fluid pressure was 100 mm H_2_O (1 mm H_2_O = 0.0098 kPa); the cerebrospinal fluid was clear; and the white blood cells, chloride, sugar, and protein were all negative, as was the cerebrospinal fluid smear (Gram stain + ink stain) and cerebrospinal fluid culture. Metagenomic sequencing of pathogenic microorganisms in the cerebrospinal fluid detected *A. flavus* (60 reads) and *Aspergillus niger (A. niger*, detected sequence number 409 reads).

After readmission to the hospital, the patient was administered meropenem 20 mg/kg q8h, linezolid 10 mg/kg q8h, and voriconazole 8 mg/kg q12h as anti-infective therapy. On the 4th day after readmission, the patient experienced vomiting and was treated with mannitol 5 mL/kg for q8h. Amphotericin B liposome intravenous injection, amphotericin B intrathecal administration, and hormone therapy were administered on the 6th day of readmission. The initial dose of amphotericin B liposomes was 0.3 mg/kg, the dose administered on the 2nd day was 0.7 mg/kg, and on the third day it was increased to the maximum dose of 1 mg/kg, which was maintained for 3 weeks; at each dose, 2 mg dexamethasone was administered before intravenous amphotericin B liposome administration. Amphotericin B was administered intrathecally at 0.1 mg for the first time and maintained at 0.5 mg after increasing by 0.1 mg each time. It was administered twice a week for a total of 2.5 mg and was administered in parallel with 1 mg dexamethasone for each intrathecal administration. The liver and kidney functions and electrolytes were monitored regularly, and potassium chloride tablets were administered to prevent hypokalemia.

The patient was hospitalized for 4 weeks with normal body temperature, occasional cough, reduced mass on the head, and improved condition, and was discharged. After discharge, she continued to take voriconazole 0.2 g q12h (8 mg/kg q12h). Regular review of liver and kidney functions, electrolytes, and blood routines showed normal results, and regular therapeutic drug monitoring of voriconazole was performed, with a serum trough concentration maintained at 2.62 to 4.5 mg/L (target range: 2–6 mg/L) after discharge. reexamination of head CT and chest CT 1 month after discharge showed that the lesions were more significantly reduced (Figs. 2 and 4), and the dose of voriconazole was adjusted to 0.175 g q12h (approximately 6.5 mg/kg q12h) to continue oral administration. Follow-up was continued until July 2021, at which point the patient had no fever or worsening cough.

## 3. Discussion and conclusions

*Aspergillus* is an opportunistic pathogen that is widely distributed in nature and is a parasitic bacterium of the upper respiratory tract. *Aspergillus* can invade the skin, mucous membranes, lungs, brain, eyes, ears, paranasal sinuses, gastrointestinal tract, nervous system, bones, and other parts. The CNS is affected secondary only to the lungs, and severe cases can cause systemic infections and death. The most common pathogen in IA is *Aspergillus fumigatus*, followed by *A. flavus, A. niger*, and *Aspergillus terreus*.^[6]^ IA mainly occurs in hosts with weakened immune function, such as patients with haematological malignancies, hematopoietic stem cells or solid organ transplantation, congenital or acquired immunodeficiency, and those who take glucocorticoids and other immunosuppressive drugs. In children with leukaemia, the main risk factor for IA is severe neutropenia secondary to chemotherapy.^[4]^

We used “pulmonary” and “central nervous system” and “aspergillosis” and “leukaemia” as keywords or medical subject heading, restricted to “all children: 0 to 18 years old,” to perform a search on PubMed. As a result, we found 8 articles reporting 9 children with invasive pulmonary and CNS aspergillosis (Table 1).^[7–14]^ Cases of 5 boys and 4 girls (1.5–14 years old) were included: 8 cases were acute lymphocytic leukaemia and one case was acute myeloid leukaemia. Among them, 5 cases were confirmed to be *Aspergillus fumigatus*, one case was combined with *A. niger*, and 4 cases were unidentified *Aspergillus* species. In our case, the child was infected with *A. flavus* and *A. niger*. Among the 10 children with leukaemia, including our case, IA occurred during the period of neutropenia.

**Table 1:** Cases of pulmonary and central nervous system aspergillosis in children with Leukemia.

Serial No.	Reference	Sex/Age (yr)	Underlying disease	Diagnostic method	Aspergillosis	Chest CT or chest radiography	Type of CNS infection	Antifungal therapy	Surgery	Duration of antifungal therapy	Outcome
1	Amanati A et al^[7]^	M/1.5	B ALL	BAL culture, Brain biopsy	*Aspergillus fumigatus, Aspergillus niger*	Small ground-glass opacities in the right and left hemithorax	Multiple brain abscesses	LAMB, voriconazole, caspofungin	External drainage	108 d	Died
2	De Leonardis F et al^[8]^	F/3	B ALL	Serum and CSF galactomannan	*Aspergillus*	A fungal ball with the air crescent sign in the left upper lobe	Brain abscess	Voriconazole LAMB + Isavuconazole	NA	161 d	Improved
3	Broenen E et al^[9]^	M/8	ALL	Serum galactomannan, sputum culture	*Aspergillus fumigatus*	5 round densities and a cavity resembling an air crescent sign	Multiple brain abscesses	Voriconazole + LAMB	Drainage of one abscess	477 d	Improved
4	Broenen E et al^[9]^	F/2.5	ALL	Serum and BAL galactomannan, BAL PCR, BAL culture, CSF culture	*Aspergillus fumigatus*	Glass aspect areas in both lungs, a halo sign in the right upper lobe	Multiple brain abscesses	Voriconazole	Trepanat-ion of the occipital lesion	720 d	Improved
5	Henze G et al^[10]^	M/12	ALL	Sputum culture; abscess pathology	*Aspergillus fumigatus*	Multiple disseminated granulomatous foci	Brain abscess	Amphotericin B,5 FC, AMB instillations into the wound cavity	Resected abscess	53 d	Improved
6	Muda Z et al^[11]^	F/10	AML	Lung histopathology, CSF PCR	*Aspergillus*	3 fungal balls	Brain abscess	LAMB+ caspofungin	Yes	42 d	Died
7	Pearson AD et al^[12]^	F/4	ALL	Autopsy	*Aspergillus*	Extensive consolidation of the right lung and a small pleural effusion	The right cerebral artery infarct with a fungal thrombus	NA	NA	NA	Died
8	Athanassiadou F et al^[13]^	M/2	B ALL	BAL culture, Serum and Sputum PCR	*Aspergillus fumigatus*	NA	Brain abscess	LAMB + voriconazole	NA	240 d	Improved
9	Prakash G et al^[14]^	M/14	B ALL	Serum galactomannan, Lung biopsy	*Aspergillus*	Bilateral multiple nodules surrounded by halo	Multiple brain abscesses	AMB	NA	9 d	Died
	The current case	F/8	ALL	BAL and CSF metagenomic sequencing	*Aspergillus flavus, Aspergillus niger*	Flaky high-density shadows with cavities, 2 caviting lung nodules with crescent sign	Multiple brain abscesses	Voriconazole + LAMB, intrathecal injection of amphotericin B	NA	240 d	Improved

5 FC = 5 Flucytosine, ALL = acute lymphoblastic leukemia, AMB = amphotericin B, AML = acute myelogenous leukemia, B ALL = B acute lymphoblastic leukemia, BAL = broncho alveolar lavage, CNS = central nervous system, CSF = cerebrospinal fluid, CT = computed tomography, F = female, LAMB = liposomal amphotericin B, M = male, NA = not available, PCR = indicates polymerase chain reaction.

The clinical manifestations of invasive pulmonary *Aspergillus* include fever and cough, with the development of chest pain and hemoptysis as the disease progresses. IPA can be classified as vascular or airway invasive. The chest CT imaging of vascular IPA demonstrates a dense, well-defined lesion, with or without halo signs, air crescent signs or cavities in nodules or consolidation lesions, and wedge-shaped, segmental, or large-lobed solid change.^[15]^ When *Aspergillus* invades the alveolar and bronchiolar walls, imaging can show non-characteristic changes, such as peribronchial consolidation, bronchiectasis, central lobular nodules, tree buds, and ground-glass changes, which are more commonly observed in the early stage of onset, and these radiographic changes represent airway invasion by *Aspergillus*.^[16,17]^ Both manifestations can occur in 1 child. In our case, the chest CT showed scattered nodules, patches, and cords in both the lungs, with high-density shadows and visible cavities. Among the 9 children whose cases were reviewed in the literature (Table 1), one did not undergo chest imaging, 2 underwent chest X-ray examination, and 6 underwent chest CT examination. Six cases showed nodular pulmonary lesions (2 cases were spherical nodules, which were considered as fungal balls), 2 cases had halo signs, 2 cases had an air crescent sign, 2 cases had ground-glass shadow changes, one case had peribronchial consolidation, and one case showed large-scale consolidation in the lungs. Chest CT scans of the same child can show multiple manifestations.

CNS aspergillosis is usually secondary to hemorrhagic dissemination of IPA or the direct spread of an invasive sinus aspergillosis.^[18]^ Studies have shown that approximately 20% of patients with IA have CNS involvement.^[19]^ CNS aspergillosis can present as insidious, asymptomatic extrapulmonary involvement that usually occurs a few weeks after pulmonary aspergillosis (range: 5–283 days, mean: 2 weeks).^[20]^ Radiologically, CNS aspergillosis can be classified into parenchymal lesions in the cerebral lobes and meningeal lesions in the meninges. Patients with meningopathies may have aneurysms or vascular stenosis related to brain infection, cerebral infarction, or subarachnoid hemorrhage.^[21]^ The main pathological manifestations of CNS aspergillosis are brain abscesses and granulomatous changes. The typical pathological feature of brain MRI is circular enhancement. In this case report, an enhanced MRI of the brain showed multiple abnormal signal lesions in the left parietal lobe with annular enhancement, and the edge of the left top subcutaneous mass with obvious enhancement. We considered the formation of multiple abscesses. Among the 9 children whose cases were reviewed in the literature (Table 1), 8 presented with brain abscesses (4 were single, 4 were multiple), and one presented with cerebral artery thrombosis and infarction.

The diagnosis of IA mainly relies on the histopathology and results of fungal culture in sterile parts. Molecular biological methods can also be used in the diagnosis of aspergillosis, and for patients with haematological malignancies and hematopoietic stem cell transplantation, GM in serum and bronchoalveolar lavage (BAL) can be used as an accurate diagnostic marker for IA. The (1, 3)-β-D-glucan detection test can also be used for diagnosis, but it is not specific for *Aspergillus*.^[1]^ Some advanced methods can also be used for diagnosis, such as pathogenic microbial metagenomic sequencing. Among the 9 children whose cases were reviewed in the literature (Table 1), 5 were diagnosed by sputum, BAL, and cerebrospinal fluid specimen culture methods; one was diagnosed by autopsy; one was diagnosed as positive by blood and cerebrospinal fluid GM test; lung histopathology and cerebrospinal fluid-PCR were positive in one case diagnosed; and one case was diagnosed by lung biopsy. In our case, *A. flavus* and *A. niger* were detected by BAL and cerebrospinal fluid (CSF) metagenomic sequencing. At present, metagenomic sequencing can be used to detect aspergillosis, which provides a more convenient method for an early diagnosis of aspergillosis. Wilson et al^[22]^ reported metagenomic sequencing of the CSF of 7 patients with meningitis, and fungal infections were detected in 4 cases, including *Cryptococcus neoformans, Histoplasma capsula, Aspergillus oryzae*, and *Candida dubriella*. As a fast and high-throughput pathogen DNA sequencing technology, metagenomics can be used to detect fungal infections. Metagenomics has a higher detection efficiency than traditional pathogen detection methods, but report interpretation needs to be combined with clinical analysis.

Currently, the drugs recommended for the treatment and prevention of IA include triazoles (itraconazole, voriconazole, posaconazole, and isavuconazole), amphotericin B, and echinocandins (mika fungin or caspofungin).^[1]^ Since 2002, voriconazole has been the preferred drug for invasive *Aspergillus* disease.^[23]^ Voriconazole is a first-line treatment for invasive aspergillosis.^[24]^Voriconazole is beneficial in preventing the recurrence of *Aspergillus* infection, and the effective rate of surgery combined with voriconazole in the treatment of invasive *Aspergillus* disease is as high as 35%.^[25]^ Voriconazole is also the primary treatment for CNS aspergillosis because of its strong ability to penetrate the blood-brain barrier. Amphotericin B lipid formulations can be used to treat patients who are intolerant or resistant to voriconazole.^[1]^ Among the 10 children, including our case (Table 1), 9 received antifungal treatment, with a treatment course of 9 to 720 days. Four patients died, and the condition of 6 patients improved; among those 6 children, 4 were treated with voriconazole or isaconazole combined with amphotericin B or liposomal amphotericin B, one was treated with voriconazole alone, and one was treated with amphotericin B combined with 5-fluoro-cytosine treatment. One patient underwent surgical drainage, one underwent surgical removal of the abscess, and one underwent surgical head drilling. According to reports, although anti-tumor drugs can cause bone marrow suppression, active surgical interventions for local lesions are acceptable.^[26]^ The Infectious Diseases Society of America guidelines recommend that for patients with CNS *Aspergillus* infection, surgical drainage and removal of infected tissues should be performed together with systemic antifungal therapy.^[1]^ However, no surgical intervention was performed in our case because of personal factors. The patient was initially treated with voriconazole when she was diagnosed with pulmonary aspergillosis. The CNS aspergillosis was developed during oral voriconazole maintenance treatment, voriconazole resistance should be noted, which was then combined with intravenous amphotericin B liposome injection and amphotericin B intrathecal administration for CNS aspergillosis. The use of amphotericin B intrathecal injection in the treatment of fungal infections remains controversial. Chemical meningitis or arachnoiditis may occur due to intrathecal injection of amphotericin B, which is related to the neurotoxic effect of amphotericin B. Numerous reports have described the successful treatment of cryptococcal meningitis with intrathecal injection of amphotericin B,^[27,28]^ and there are few reports on the treatment of aspergillosis by intrathecal injection of amphotericin B. In the current case, the low dose of amphotericin B was injected intrathecally, and no obvious adverse reactions were observed. reexamination of the head and chest imaging showed that the absorption of the lesions was increased, indicating that the combined intrathecal injection of amphotericin B in the treatment of invasive *Aspergillus* achieved a good effect. Given the lack of related reports, further clinical studies are needed to confirm the specific dosage and efficacy.

With the continued improvement of clinicians’ understanding of invasive lung and CNS aspergillosis in children, early diagnosis and treatment are crucial for prognosis of the disease. Presently, pathogenic diagnosis of aspergillosis is difficult, and metagenomic sequencing is expected to become an important supplement to the pathogenic diagnosis of aspergillosis. Voriconazole remains the drug of choice for IA and can be combined with amphotericin B or liposomal amphotericin B.

## Acknowledgments

We wish to thank the patient and her family for agreeing to publish this paper. This study was approved by the ethics committee of our hospital. We would like to thank Editage (www.editage.cn) for English language editing. We are grateful for the financial support from the “Tianjin Medical Key Discipline (Specialist) Construction Project”.

## Author contributions

**Conceptualization:** Yingxue Zou.

**Data curation:** Jia Zhai.

**Formal analysis:** Run Guo.

**Methodology:** Xingnan Jin.

**Supervision:** Yingxue Zou.

**Validation:** Yingxue Zou.

**Writing – original draft:** Dongmei Meng.

**Writing – review & editing:** Jiao Li.
